# Modeling Geospatial Patterns of Late-Stage Diagnosis of Breast Cancer in the US

**DOI:** 10.3390/ijerph14050484

**Published:** 2017-05-05

**Authors:** Lee R. Mobley, Tzy-Mey Kuo, Lia Scott, Yamisha Rutherford, Srimoyee Bose

**Affiliations:** 1 School of Public Health and Andrew Young School of Policy Studies, Georgia State University, 1 Park Place, Atlanta, GA 30304, USA; 2 Lineberger Cancer Center, University of North Carolina at Chapel Hill, Chapel Hill, NC 27599, USA; tkuo@email.unc.edu; 3 School of Public Health, Georgia State University, Atlanta, GA 30304, USA; lscott19@student.gsu.edu (L.S.); yrutherford1@student.gsu.edu (Y.R.); sbose3@student.gsu.edu (S.B.)

**Keywords:** late-stage cancer diagnosis, breast cancer, residential isolation, health disparities, geographic heterogeneity

## Abstract

In the US, about one-third of new breast cancers (BCs) are diagnosed at a late stage, where morbidity and mortality burdens are higher. Health outcomes research has focused on the contribution of measures of social support, particularly the residential isolation or segregation index, on propensity to utilize mammography and rates of late-stage diagnoses. Although inconsistent, studies have used various approaches and shown that residential segregation may play an important role in cancer morbidities and mortality. Some have focused on any individuals living in residentially segregated places (place-centered), while others have focused on persons of specific races or ethnicities living in places with high segregation of their own race or ethnicity (person-centered). This paper compares and contrasts these two approaches in the study of predictors of late-stage BC diagnoses in a cross-national study. We use 100% of U.S. Cancer Statistics (USCS) Registry data pooled together from 40 states to identify late-stage diagnoses among ~1 million new BC cases diagnosed during 2004–2009. We estimate a multilevel model with person-, county-, and state-level predictors and a random intercept specification to help ensure robust effect estimates. Person-level variables in both models suggest that non-White races or ethnicities have higher odds of late-stage diagnosis, and the odds of late-stage diagnosis decline with age, being highest among the <age 50 group. After controlling statistically for all other factors, we examine place-centered isolation and find for anyone living in an isolated Asian community there is a large beneficial association (suggesting lower odds of late-stage diagnosis) while for anyone living in an isolated White community there is a large detrimental association (suggesting greater odds of late-stage diagnosis). By contrast, living in neighborhoods among others of one’s own race or ethnicity (person-centered isolation) is associated with greater odds of late-stage diagnosis, as this measure is dominated by Whites (the majority). At the state level, living in a state that allows unfettered access to a specialist is associated with a somewhat lower likelihood of being diagnosed at a late stage of BC. Geographic factors help explain the likelihood of late-stage BC diagnosis, which varies considerably across the U.S. as heterogeneous compositional and contextual factors portray very different places and potential for improving information and outcomes. The USCS database is expanding to cover more states and is expected to be a valuable resource for ongoing and future place-based cancer outcomes research.

## 1. Introduction

Cancer is the second most common cause of death in the U.S. [1,2], and breast cancer (BC) is the most common cancer in women [1,2,3]. Mammography screening rates are lower than recommended, resulting in higher rates of late-staged breast cancers and higher morbidity and mortality rates [1,4,5]. If the cancer has spread to the regional lymph nodes, the 5-year survival rate is 85%. If the cancer has spread to a distant part of the body, the 5-year survival rate is 26% [6]. About 35% of new breast cancers (BCs) in the U.S. between 2009–2013 were diagnosed at a late stage (29% regional, and 6% distant), where morbidity and mortality burdens are higher [5]. Of policy importance, there are disparities across population racial or ethnic subgroups in the likelihood of BC being diagnosed at late-stage [4,5].

**Figure 1:**
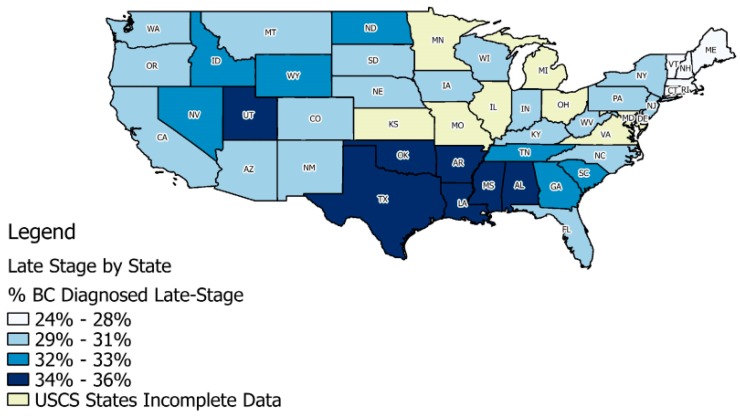
Proportions of breast cancer (BC) cases diagnosed at late-stage (defined as regional and distant) in the U.S., U.S. Cancer Statistics (USCS) database 2004–2009.

The overall rates of late-stage diagnoses for BC in the U.S. vary considerably across the states (Figure 1), based on data for 2004–2009 from 40 states in the United States Cancer Statistics (USCS) database. Included are the 40 states that provided county identifiers to use in multivariate modeling and full registry population data during 2004–2009; others are labeled as “USCS States Incomplete Data”. The database is a population-based surveillance system of cancer registries with data representing 99% of the U.S. population [5,7].

Newly diagnosed BC cases during 2004–2009 in Figure 1 exhibit patterns in state-level proportions of these late-stage diagnoses that vary across the U.S., where the mapping uses the quantiles of the distribution of rates across states, and states with proportions above the national average are shaded as the darkest two colors. In these data, the national average for the proportion of BC diagnosed at late stage is 31%, and states with the worst outcomes are mostly in the Middle South and Utah. Utah was the last state in the U.S. to pass a state mandate requiring private insurers to cover mammograms [8]. Since 2010, a Federal Mandate under the Affordable Care Act requires all private insurers to cover screening mammograms with no out-of-pocket costs to consumers.

A large literature (described below) has examined the role that social forces may play in shaping health outcomes such as these, where in addition to availability of services and financial means, personal information and motivation are required to enable timely access of preventive breast cancer screenings and knowledge regarding BC risk factors or etiology of rare breast cancers not found by mammography. We focus here on the role that residential segregation may play in providing this sort of support.

### Literature on Residential Segregation and Health

Williams and Collins [9] argued that residential segregation caused racial or ethnic disparities in health outcomes, because it determined access to education and employment opportunities and thereby caused differences in socioeconomic status (SES). Lower personal or community SES has been associated with worse health outcomes in hundreds of studies, and is seen as a fundamental cause of health disparities. Researchers have studied the residential segregation phenomenon using various measures. Many adopt measures proposed by Massey and Denton [10], who rigorously defined several measures as a multidimensional phenomenon. The measures described by Massey and Denton [10] varied along five distinct axes: evenness, exposure, concentration, centralization, and clustering. Examples of these dimensions are found in measures such as the Diversity Index (evenness), Isolation Index (exposure), Interaction Index (exposure), Index of Spatial Proximity (clustering), and White’s Clustering Measure (clustering).

Kramer and Hogue [11] reviewed 39 studies of ecological factors and social outcomes, determining that isolation, clustering, and dissimilarity indices had been used most often. In this study, we use the Isolation Index, which is a minority-weighted average across census tracts in each county, using the formula [10]:

$$I_{j}=\overset{N}{\underset{i=1}{\sum }}\frac{x_{i}}{X}\frac{x_{i}}{t_{i}}$$

Numerous census tracts are nested within each county. The Isolation Index *I* for county *j* is defined as follows: $x_{i}$ is the number of people in a minority group in tract *i*; *X* is the sum of all members of that minority group across all tracts in county *j*; $t_{i}$ is the total number of all people in tract *i*; *N* is the number of tracts within each county *j*. The county Isolation Index defined in this way for a particular minority group reflects the extent to which the minority group comes into contact with others of this minority group within the county under random mixing of individuals within the county. The index ranges in value from 0 to 1, and a higher index value reflects the higher probability of contact among members of the minority group (high segregation), and thus lower probability of contact among the minority group and people of other races or ethnicities.

Studies of late-stage breast cancer outcomes and cancer screening behavior have mostly used the Isolation Index as a measure of residential segregation [12,13,14,15,16,17,18,19]. The Isolation Index has been interpreted by some as measuring social cohesion or support when defined at the neighborhood level. However, some studies argue that residential isolation reflects an adverse environment [9,12,20,21,22,23]. Others argue that social support might be positively enhanced by a high degree of clustering into enclaves which enhances political empowerment [17,24,25,26].

To date, there is no consensus in the health outcomes literature regarding whether residential isolation is a beneficial, or a harmful effect. In many previous studies, the residential Isolation Index is defined at the area level (e.g., county), and matched to the county of residence for all subjects in the model. In this way, several isolation indices, defined for several different races or ethnicities may be included simultaneously in the model. This is the approach we take in Model 1 in this paper. Thus, these variables model the effects of anyone living in a place where certain races or ethnicities are most highly segregated—what we refer to here as **a place-centered approach**.

**Figure 2:**
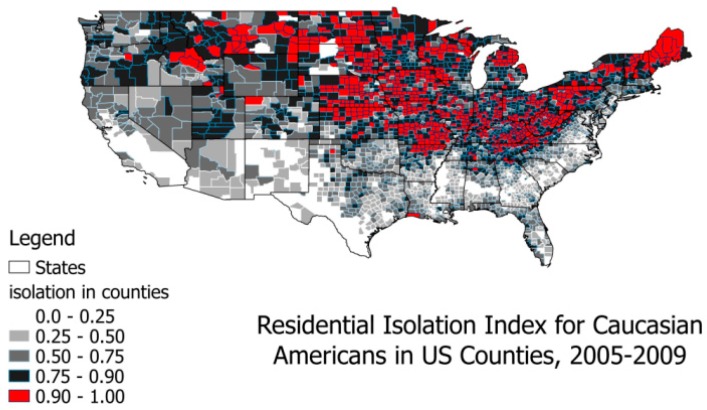
Residential isolation among Whites in U.S. counties, 2005–2009.

**Figure 3:**
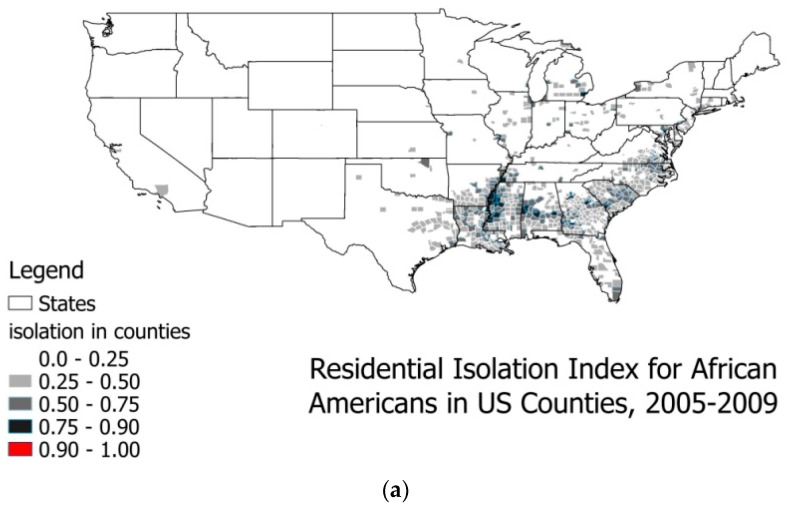

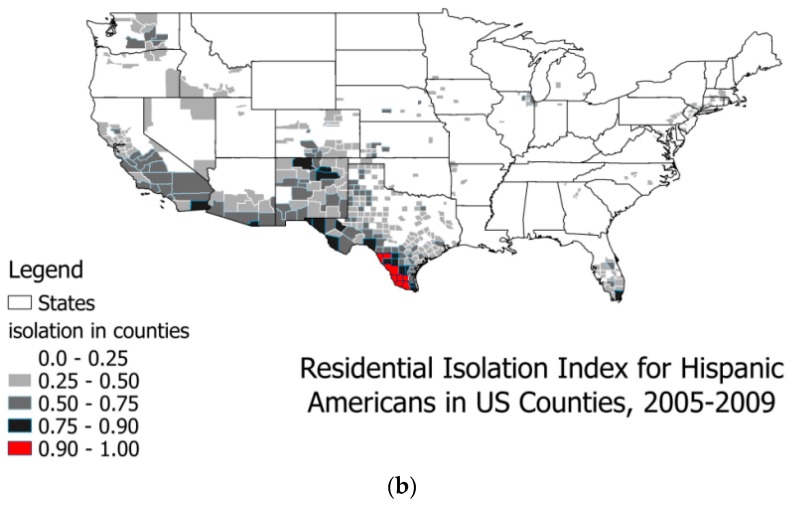
(**a**) Residential isolation among Blacks and Hispanics in U.S. counties, 2005–2009. (**b**) Residential isolation among Hispanic Americans in U.S. counties, 2005–2009.

As shown in Figure 2 and Figure 3a,b, highly segregated places by race or ethnicity exhibit distinct geospatial patterns in the U.S. Wilkes and Iceland [27] argue that hyper-segregated places are more prevalent for Blacks and Hispanics, however, this seems more prevalent for Whites in the U.S., as shown in Figure 2 and Figure 3. Defining the hyper-segregation range for the Isolation Index as attaining a value of at least 0.90, colored red in Figure 2 and Figure 3, we find hyper-segregation is met for Hispanics in only eight counties along the Rio Grande River Valley bordering Texas and Mexico, and never met for Blacks in any counties. (However, this criterion is likely to be met for Blacks at smaller spatial scales than county, such as census tracts located in some inner cities). By contrast, in the continental United States this definition of hyper-segregation is met for Whites in 771 counties, 522 of which (68%) are in our 40 study population states. We do not map the Isolation Index for Asians because it never exceeds the 0.25–0.50 (light grey) range, and that range is found for Asians in only 13 counties of the U.S.: six in California (San Francisco and San Diego Bay Areas), three in New York, two in New Jersey, one in Texas, and one in Louisiana. Similarly, we do not provide a map for the Native American Isolation Index, because it reaches hyper-segregation levels in all of the areas describing the Native American Reservation lands, but nowhere else, and the population of Native Americans with BC is very small.

At the county level in the U.S., hyper-segregation as defined here is mainly a White phenomenon (Figure 2). When interpreting the residential isolation variable’s effect in place-centered modeling, this is tantamount to predicting differential outcomes in these most highly segregated places. What this sort of modeling does *not* do is capture the effect of living in a highly segregated place of one’s own race or ethnicity, which we refer to here as a **person-centered approach**.

Three papers examining person-level predictors of late-stage BC diagnosis used a person-centered approach to measure residential isolation. More specifically, they included in modeling only the area-level Isolation Index that matched the person’s race or ethnicity [13,14,18]. The first two studies [13,14] analyzed different racial or ethnic subgroups in separate models including only their own race’s Isolation Index. The third study [18] combined all races and ethnicities together in a single model and included a single person-centered construct (as we do in Model 2 in this paper). In these papers, the isolation effect seems beneficial among the Surveillance, Epidemiology, and End Results (SEER) Registry subjects [14,18] and beneficial for California women living in the poorest communities who are Hispanic or Black [13]. The purpose of this paper is to look at a range of predictors of late-stage BC diagnoses across the U.S., with a special focus on these two different approaches to measuring residential isolation effects (place-centered and person-centered).

## 2. Population Data and Methods

This study includes cancer cases diagnosed during 2004–2009 from the United States Cancer Statistics (USCS) database, which has information on demographics (age, gender, race, ethnicity), tumor characteristics, and geographic location (U.S. county of residence) at time of BC diagnosis. Access to the database with place of residence information for cancer patients is restricted to researchers with approved research plans with analyses conducted inside secure federal Research Data Centers (RDCs). There is no access to the Internet from inside the RDC, and all results must be reviewed before they can be released from the RDC and published.

We restricted the sample to all persons having breast cancer and excluded records: when these were not their primary cancers, when records featured unknown cancer stage or unstaged cancer (~2.5% of cases), or when diagnosis was by autopsy or death certificate (<1% of all cases). These restrictions resulted in 973,143 individuals with BC living in 2366 U.S. counties located in 40 of the United States. Most states participate in the USCS registry data system, but three did not (Kansas, Maryland, Minnesota), and four states did not allow use of county of residence information (Illinois, Michigan, Missouri, Ohio). We excluded these seven states and an additional state, Virginia, because data were not available until 2007. We also excluded Hawaii and Alaska because of missing contextual data, leaving a total of 40 states included in the analysis (see Figure 1).

Counties vary widely, in terms of geographic size, across the U.S. The smallest is Loving, Texas with 83 residents in 2010, and the largest is Los Angeles, with 9.8 million in 2010. However, counties are designated geo-political units that are centers of local government which are quite stable in terms of the geospatial boundaries over time. Persons are the first level used in our analyses. Counties are the second level, and states (inside which nest the counties) are the third level. Person-, county-, and state-level data were used as predictors of the likelihood of late-stage BC diagnosis. Table 1 describes all of the variables and provides sample statistics. County-level contextual variables include average distance to closest mammography services, managed care insurance penetration, mammography screening rate, the percent population with no health insurance, the percent population living in rural areas, and the Isolation Index measures defined for Black, Asian, Hispanic, and White populations. Holding other personal and contextual factors constant statistically, the central focus is to examine the effects of residential isolation when modeled two different ways (place-centered, person-centered).

To construct the person-centered Isolation Index, we first merged the county level isolation indices for Whites, Asians, Hispanics, Alaska Native/American Indian, Pacific Islander, and Blacks into the person level file using each person’s county of residence. We then identified the pertinent area-level Isolation Index for each person, based on that person’s racial or ethnic information. For example, the race-matched (person-centered) Isolation Index for a Black would be the county level Black Isolation Index for the county of residence. Likewise, the race-matched Isolation Index for a Hispanic person living in that same county would be the county level Hispanic Isolation Index. Table 1, describing contextual characteristics of communities obtained from a variety of sources, shows that, in our data, only 1.3% of people were American Indian, Pacific Islander, or other races (combined in the “other” group). Because there was not a separate Isolation Index defined at the area level for the “other” races or ethnicities, we used the average value of the existing isolation indices to define an index to match with this “other” group.

In addition to person and county-level factors, we included one state-level regulatory variable which has the potential to impact screening behavior as well as diagnostic accuracy and heightened risk factor awareness, consequently reducing late-stage cancers. The “Direct Access to Specialist” mandate requires insurance plans to cover direct access to specialists, without referrals from their primary health plan provider [8]. This leniency to seek outside opinion could plausibly affect the availability of information regarding the importance of cancer screening, familial cancer risks, factors limiting diagnostic accuracy, and other concerns that would enable patients to better align with the best cancer screening providers and best medical advice. Ward et al. [28] found that people covered by the most preferred types of insurance (plans with the greatest freedom of choice of providers) were less likely to be diagnosed with late-stage cancer, which suggests that timely access to preferred physicians and specialists is important, and state regulations that enhance this may improve access. The regulatory variable “Direct Access to Specialist” is thus expected to enhance early-stage diagnosis outcomes, and thus be associated with lower odds of late-stage diagnoses.

## 3. Statistical Methods

This paper aims to examine various predictors of late-stage BC at first diagnosis, an outcome associated with higher morbidity and mortality and found in almost one-third of BC cases diagnosed in the U.S. [5]. Person-level, area level (county), and regional level (state) predictors are included in the multilevel model of late-stage BC diagnosis outcomes. Variables of particular interest are the Isolation Indices, which have been widely used in health outcomes studies with sometimes disparate findings. The Isolation Index is defined for each minority and reflects the degree to which their residences are segregated by race or ethnicity. A higher value of the Isolation Index for minorities indicates that the minority group has less interaction with persons of other races or ethnicities. For Whites, it reflects the situation where Whites rarely come into contact with non-Whites (minorities).

We use the Isolation Index in two different ways, contrasting two separate models, to help explain why different studies have different findings regarding the isolation effects. As defined here, the person-centered isolation measure should capture the aspect of living in more segregated communities of one’s own race or ethnicity. We anticipate that this measure would reflect some sort of social cohesion or support that would perhaps motivate appropriate cancer screenings and result in lower incidence of late-stage cancer diagnoses.

We specified a three-level random intercept logistic model for the late-stage diagnosis outcome, with patients nested in counties which are nested in states. We used a multilevel modeling framework because we wanted to fit the regression to individuals while accounting for unexplained variation among counties and states. Ignoring the county and state level effects, when they are important, is tantamount to having omitted variables in the model, which can bias individual coefficient estimates [29]. In addition, when the higher-level (e.g., county, state) covariates are of interest, failing to account for their structural similarity across individuals within them can increase their apparent statistical significance [30]. Because a robust estimate of the county-level isolation effects is the main focus of the paper, this empirical approach is warranted.

We estimated multilevel models using pooled data across the 40 states. We used the Generalized Linear Latent and Mixed Model (GLLAMM) procedure [31,32] in Stata software (StataCorp LP: College Station, TX, USA) [33] to fit models that allow both county and state intercepts to vary. To understand the differences in models using place-centered versus person-centered Isolation Index constructs, we specify two models: the place-centered one including several area-level isolation indices matched to each person’s county of residence (Model 1), and the person-centered one including the single race-matched Isolation Index from the matched person’s county of residence (Model 2).

## 4. Results

The statistics provided in Table 1 describe the study population. About 77% of the population of women diagnosed with BC in these 40 states are White, 10% are Black, 8% are Hispanic, and 3% are Asian; other groups make up about 1% of the BC cases. Almost a quarter (23%) of the BC cases are women under the age of 50, and 37% are in the 50–64 age group; only 41% of cases are among women aged 65 and older. Table 1 also includes sample statistics for the county level covariates. The Isolation Index for Whites has the largest mean, followed by the index for Hispanics. These two groups are more segregated into counties than other groups, as shown in Figure 2 and Figure 3. The average penetration of managed care insurance in counties is about 16%, with a wide variance. The average distance to closest mammography facility is 6 miles. The average percentage of people who received mammograms is about 24%, while the average percentage of the population without health insurance is about 18%. Most states (34) in the 40-state sample allowed direct access to specialist, and six states did not.

The results from statistical modeling are presented in Table 2. Only the statistically significant estimates (shown in bold type) are discussed. Person-level effects are consistent across the two models of residential isolation. Blacks (OR 1.45; 95% CI 1.428–1.473) and Hispanics (OR 1.253; 95% CI 1.231–1.275) are more likely to be diagnosed at late-stage than Whites. Asians are less likely to be diagnosed at late stage for BC (OR 0.974; 95% CI 0.949–1.000; not statistically significant in the person-centered model), as compared to Whites. The “other” races and ethnicities are less likely than Whites to be diagnosed at late stage BC. The youngest people are much more likely than older people (age 75+, the reference group) to be diagnosed at late stage (OR 1.342; 95% CI 1.323–1.361), with the age 50–64 group also showing greater odds (OR 1.063; 95% CI 1.049–1.077). For county predictors, higher area mammography screening rate is associated with lower likelihood of late-stage diagnosis, and higher managed care penetration is associated with lower late-stage BC diagnosis. Poverty, percent uninsured, and rural aspect of county of residence are significant predictors in the person-centered isolation models. The state-level regulatory variable “free choice of specialist” is associated with lower likelihood of late-stage BC diagnosis.

The isolation measures are of particular interest in this study. The place-centered model (Model 1) shows a protective (suggesting lower odds of late-stage diagnosis) effect associated with living in a highly segregated Asian community, and a detrimental (suggesting greater odds of late-stage diagnosis) effect associated with living in a highly segregated White community. Place-centered Black or Hispanic community effects are not statistically significant predictors of late-stage BC. By contrast, the person-centered isolation model (Model 2) shows a small detrimental effect from living in segregated communities of one’s own race or ethnicity.

One advantage of using a multilevel model is the ability to determine the variance components at each level. The variances at the county and state levels are very small (bottom rows Table 2). As noted in previous statistical studies, these small variance components suggest that the model fit is good. Gumpertz et al. [34] used a random intercepts formulation for the multilevel model (with person and area-level covariates to model advanced-stage breast cancer incidence in Los Angeles county). As described in Gumpertz et al. [34] and in Oakes [29], a small variance estimate for the area-level random effect indicates that the contextual factors included in the model well account for geographic heterogeneity in the explanatory factors. However, these variance components reflect overall model fit and may mask the fact that model fit is not as good in some places as it is in others.

To determine whether there were places where the model fit was problematic, either in terms of over-predicting or under-predicting the late-stage BC diagnosis rate in the county, or in terms of spatial correlation of the unexplained variance across counties, we did the following ad-hoc analyses. For each logistic regression model, we obtained predicted values from the regression, which include the random intercepts components. Next, we aggregated these predictions across individuals and calculated the average prediction at the county level. As the comparator, we aggregated the actual person-level binary indicator of late-stage diagnoses to the county level, yielding the actual observed proportion. We then compared the observed proportion to the average probability predicted by the model, and calculated differences. We noted several counties that were large outliers in each tail of the differences distribution, but simple mapping of the residuals showed no spatial pattern. This spatial randomness was confirmed by the global Moran’s I test of spatial autocorrelation.

The global Moran’s I statistic is a formal test for spatial clustering of any variable across the landscape [35]. Possible values of the test statistic range from −1 to +1, with −1 indicating perfect dispersion, values near 0 indicating spatial randomness, and values near +1 indicating perfect clustering. Dispersion would mean that dissimilar counties were located together, while clustering would manifest as spatial groupings of highly similar counties. The calculated test statistics for each model were very small (<0.01), suggesting no statistical evidence of spatial autocorrelation in the county-level differences, for either model. Thus, we concluded that the model specification may have adequately explained the spatial heterogeneity in the sample and left no unexplained variation that is correlated across the counties.

## 5. Discussion

The person-level factors all have the expected associations with the outcome. Besides the isolation indices, all other area predictors are significant and have the expected direction of association—managed care penetration, area screening rate (mammogram use), percent uninsured, rural residence, living in poverty, and state insurance mandate. Of central focus in this paper are the area-level residential isolation indices.

The results show that the place-centered isolation model (Model 1), reflecting segregation in communities by different racial or ethnic groups, reflects something very different than the person-centered isolation variable. The place-centered isolation indices pick up effects of living in geographically disparate, highly segregated places (Figure 2 and Figure 3). These types of places are entered independently into the model, and differences in associations with the likelihood of late-stage BC diagnosis are noted. Apparently, those people living in highly segregated Asian places (mainly the California Bay Areas and New York City) have reduced likelihood of late-stage BC diagnosis. Living in highly isolated Black or Hispanic communities has no significant association with the outcome, while living in highly segregated White communities is associated with greater likelihood of late-stage BC diagnosis.

The finding that women living in highly isolated Asian communities have lower likelihood is not surprising, because Asians have lower likelihood of late-stage BC diagnosis compared to Whites. However, the null findings for the isolated Black and Hispanic communities suggest a more complex scenario. These groups are much more likely than Whites to be diagnosed at late-stage, however, living in highly segregated Black or Hispanic communities is not associated with greater risk. Perhaps concerted efforts to target these communities and intervene to reduce disparities can explain how these places seem to be neutral risk environments. The most puzzling fact is that residents of highly isolated White communities are at greater risk of late-stage diagnosis, while White women have relatively lower risk of such diagnosis. Using two different approaches to measuring residential isolation effects, we expected that the person-centered measure (Model 2) would reflect some sort of social cohesion or support that would perhaps improve information regarding relative risks and motivate appropriate cancer screenings, resulting in lower incidence of late-stage cancer diagnoses. This expectation was not met in the person-centered model (Model 2). Women living in counties more highly segregated by their same race or ethnicity exhibited a significantly higher likelihood of being diagnosed with late-stage BC. Because the person-centered isolation measure picks up the effects of White isolation in addition to other races or ethnicities, it provides a net effect estimate of the residential association with people of one’s race, which is dominated by Whites (the vast majority).

As shown in Figure 2, the most highly isolated White areas are not predominately an Appalachian phenomenon, as they do not cluster along the Appalachian Mountain range that stretches across the Eastern Seaboard from the South to the North East. Correlation analysis (not shown) found that the most highly isolated White communities were more likely to be rural, but less likely to be poor. The isolated but *poor* rural White communities (found throughout Appalachia) had a higher demographic diversity score, and a somewhat lower White isolation measure. These findings suggest there may be a widespread problem in highly isolated White communities, which are perhaps an understudied “reverse disparity” bearing further thought and future investigation.

This finding of a detrimental White isolation effect is consistent with recent reports of a rise in U.S. mortality rate that is being driven by rapidly increasing White mortality [36,37]. Social scientists studying cancer control have proposed theories related to how “relative risk perception” influences behaviors such as health-seeking and preventative measures [38,39,40]. These scientists have argued that a fundamental problem in many poor, isolated, rural areas (focusing on the Appalachian region of the U.S.) is that competing life stressors take precedence over concerns regarding personal health, culminating in a sense of denial or reluctance to recognize health risks for fear of “asking for trouble”. Thus, in poorer rural communities, there is probably lower interest in pursuing information that would enable timely utilization of preventive breast cancer screenings, or seeking information about BC risk factors or etiology of rare breast cancers not found by mammography.

The “perception of risk” framework may well explain health disparities in poorer rural communities, but does not explain the widespread isolated rural White communities that are not poor, identified as problematic in our study. We searched the literature for alternative theories that might explain an apparent aversion to being well-informed or for neglecting one’s health. Cherlin [36,37] proposed a theory based on an older literature to explain the rise in White mortality—the “reference group theory”—pioneered by the social psychologist Herbert H. Hyman in 1942, and further developed by the Columbia sociologist Robert K. Merton in the 1950s [41]. According to this theory, a person’s behavior is governed by how one perceives their standing relative to others. In recent decades, Whites have gained less relative to their parents while minorities have gained more, which has eroded the relative position of Whites, and may explain why Whites, who actually have more in the U.S. than minority groups may feel that they are losing ground/have less. This sense of pessimism can lead to despair and a sense of failure, which can manifest in unhealthy behaviors and a fatalistic attitude. This notion is supported by the General Social Survey conducted by the University of Chicago, which asks Americans to compare their standard of living to that of their parents. In 2014, among 25- to 54-year-olds without college degrees, Blacks and Hispanics were much more positive than Whites. This represents a reversal from 2000, when Whites were more positive than Blacks [36]. There may be other explanations, and hopefully further research will elucidate them.

One potential limitation of these data is their lack of timeliness. Because of about a 4-year lag in availability of the data in the USCS database, and the lengthy request and approvals process required to obtain permission to analyze it, it would not be possible to use these data for real-time population surveillance. Because this RDC system of data support is relatively new, we expect that the lag time will shorten, and that more timely analyses will be possible in the future. The USCS is also expanding to include more states over time, perhaps to include all states in the U.S. in the future. Future research can validate whether our findings here for the U.S. during 2004–2009 remained consistent over more recent time periods.

## 6. Conclusions

The U.S. health outcomes literature has focused extensively on disparities in health outcomes among persons of different racial, ethnic, gender, or age groups. The focus has largely been on minority disparities relative to Whites, as person-level differences often suggest a White advantage. In this paper, a comprehensive set of person, area-, and regional factors were considered as predictors of the likelihood of late-stage BC diagnoses among a large population of women with BC residing in the U.S. This population affords the opportunity to examine racial or ethnic, as well as age-related disparities and whether aspects of place of residence are important predictors. As regards place, one focus of this paper was to examine two different approaches to the use of residential segregation factors in modeling. The literature has largely used the first approach, and limited the investigation to isolated minority community effects. In this paper, we expand the focus to include White community effects, and introduce a new approach that is more person-centered than the past literature. The results contrast these two different approaches to measuring residential segregation effects, and the discussion centers on how the effect estimates in Models 1 and 2 have very different interpretations.

The modeling approach (MLM with random intercepts) is one that limits bias from omitted variables in model specifications [29], and is robust to differences in population sizes across areas [30]. The modeling also captures quite well the heterogeneity in factors across the landscape of the 40 states studied. The findings are therefore quite robust, however, more research is needed to fully understand the social implications.

While the main focus of the paper was on place-based social aspects in the prediction of late-stage BC diagnoses, we cannot ignore our person-level findings. At the person level, findings were consistent with other studies. Blacks and Hispanics were more likely than Whites to be diagnosed at late-stage of BC, while other races or ethnicities were less likely. The likelihood of being diagnosed at late stage was highest among the youngest group (<age 50) to whom screening guidelines are less focused. This younger group is more likely to experience rarer forms of BC, those that are quite aggressive and/or cannot be reliably detected by mammogram. Rare forms of BC are now receiving greater attention in funded National Institutes of Health (NIH) studies, and perhaps ongoing research using the USCS database will enlighten us regarding the etiology of these cancers so that greater prevention will be possible. Other factors in general that may explain the likelihood of being diagnosed at a late stage for BC in future work may include environmental contaminants, marital status, parity, cultural or social factors that impact risk factor awareness, cognizance of familial risk, awareness of importance of breast cancer screening, diagnostic accuracy, or willingness to seek expert advice about breast health. Other place-based factors such as area BC screening and managed care penetration were additional significant predictors of late stage BC diagnoses, and both were found to reduce the likelihood of late-stage BC diagnosis. A state insurance mandate that required unfettered access to specialists was found to be protective. In the vastly heterogeneous U.S., these place-based factors are helpful in predicting variation in the odds of late-stage BC diagnosis. These results provide definitive insights into both where and why disparities in late-stage diagnosis of BC in the U.S. may exist, yet more work is definitely needed in understanding disparities in the incidence of BC and in the incidence of late-stage diagnoses of BC. The USCS database is an excellent resource to enhance comprehension of these important disparities.

## Figures and Tables

**Table 1:** Multilevel Model Variables: Description, Rationale, and Sample Statistics.

Variable (Units of Measure)	Rationale for Inclusion	Sample Statistics	
		Mean (Proportion)	Standard Deviation
**Outcome**: whether cancer patient was diagnosed at a late stage (regional or distant = 1, else = 0)	Late stage diagnosis is indicative of lack of knowledge regarding personal cancer risk, or the importance or availability of screening; lack of timely or proximate access to services, lack of funds to pay for, and cultural or other barriers related to utilization of timely cancer screening	0.308	0.461
**Person-level predictors**			
African American	The national statistics cite African Americans as a disadvantaged group, with higher likelihood of late-stage BC than whites, the reference group	0.101	0.301
Hispanic	The national statistics cite Hispanics as a disadvantaged group, with higher likelihood of late-stage BC than whites, the reference group	0.081	0.273
Asian	The national statistics cite Asians as an advantaged group, with lower likelihood of late-stage BC than whites, the reference group	0.033	0.178
White (reference category)	The reference group	0.773	0.419
Race all others		0.013	0.112
age < 50	BC screening protocols recommend to start screening at age <50 for high risk individuals	0.226	0.223
age 50–64	BC screening protocols recommend to start screening at age 50 for average risk individuals; this is the prime age bracket for screening	0.366	0.397
age 65–74	Medicare insurance coverage begins at age 65 for people who are eligible for Social Security benefits	0.219	0.425
age 75+ (reference category)	Screening is not needed or recommended as often for older individuals who have had regular screening at younger ages	0.189	0.463
**County-level predictors**			
Isolation index white	This index reflects the degree to which whites are proximate to other whites in their county of residence (2000)	0.774	0.144
Isolation index African American	This index reflects the degree to which African Americans are proximate to other African Americans in their county of residence (2000)	0.101	0.301
Isolation index Hispanic	This index reflects the degree to which Hispanics are proximate to other Hispanics in their county of residence (2000)	0.216	0.203
Isolation index Asian	This index reflects the degree to which Asians are proximate to other Asians in their county of residence (2000)	0.073	0.086
Person-centered isolation measure	This constructed measure matches the area-level Isolation Index to the person based on race or ethnicity, and reflects the degree to which people are proximate to others of their same race or ethnicity (2000)	0.716	0.225
Managed care penetration (%)	Managed care has transformed the way medicine is practiced in highly-penetrated markets, with preventive care services more prevalent/utilized more intensively (2005)	15.9	14.7
Distance (miles)	Calculated as the average distance (miles) over all ZIP codes with centroid in the county to *closest* mammography provider ZIP code. Greater distance to closest provider of BC (mammogram) screening suggests impeded access to preventive care services. Based on 100% Fee for Service (FFS) Medicare utilization of mammography services (2006)	6.02	6.10
Screening rate (%)	Percent of the 100% FFS Medicare population residing in the county and alive all year that utilized cancer screening (mammography) (2006)	23.60	3.18
Percent uninsured (%)	% of the under-age-65 population who did not have health insurance (2005)	17.73	5.45
**State-Level Policy Variable** Direct Access to Specialist (1 = yes, 0 = no) in 2004	Access to a specialist without need of referral from a primary care physician may result in better matching and more timely care. Hypothesized to increase access for less well insured individuals or those in more stringent managed care plans. Younger people tend to be enrolled in these more stringent managed care plans, which are less costly but restrict access and choice. Source: NCSL, 2010	Six out of the 40 states did not have Mandate: ND, SD, NE, IA, WY, OK	

**Table 2:** Multilevel Models Specified with Two Different Segregation Measures as Predictors of Late-Stage Diagnosis of BC.

Variables	Model 1: Place-Centered Isolation				Model 2: Person-Centered Isolation				
	Odds Ratio	P > |z|	Lower CI	Upper CI	Odds Ratio		P > |z|	Lower CI	Upper CI
**Person-level variables. Reference groups white, age > 74**									
Black	**1.45**	**0.00**	**1.43**	**1.47**	**1.48**		**0.00**	**1.45**	**1.51**
Hispanic	**1.25**	**0.00**	**1.23**	**1.28**	**1.27**		**0.00**	**1.25**	**1.30**
Asian	**0.97**	**0.05**	**0.95**	**1.00**	0.99		0.91	**0.97**	**1.03**
other	**0.88**	**0.00**	**0.84**	**0.92**	**0.90**		**0.00**	**0.86**	**0.94**
age <50	**1.34**	**0.00**	**1.32**	**1.36**	**1.34**		**0.00**	**1.32**	**1.36**
age 50–64	**1.06**	**0.00**	**1.05**	**1.08**	**1.06**		**0.00**	**1.05**	**1.08**
age 65–74	**0.89**	**0.00**	**0.88**	**0.91**	**0.89**		**0.00**	**0.88**	**0.91**
**County-level variables**									
distance closest provider	1.00	0.63	1.00	1.00	1.00		0.11	1.00	1.00
distance squared	1.00	0.75	1.00	1.00	1.00		0.50	1.00	1.00
isolation Black	1.01	0.58	0.97	1.07	.		.	.	.
isolation Asian	**0.75**	**0.00**	**0.68**	**0.84**	.		.	.	.
isolation Hispanic	1.02	0.54	0.95	1.10	.		.	.	.
isolation White	**1.18**	**0.00**	**1.06**	**1.30**	.		.	.	.
person-centered isolation	.	.	.	.	**1.06**		**0.00**	**1.02**	**1.10**
managed care penetration	**0.90**	**0.00**	**0.84**	**0.96**	**0.82**		**0.00**	**0.77**	**0.87**
area screening rate	**0.98**	**0.00**	**0.97**	**0.98**	**0.98**		**0.00**	**0.98**	**0.98**
percent uninsured	1.00	0.92	1.00	1.00	**1.01**		**0.00**	**1.00**	**1.01**
percent rural residence	1.04	0.18	0.98	1.10	**1.06**		**0.00**	**1.02**	**1.10**
percent poverty	**1.01**	**0.00**	**1.01**	**1.01**	**1.01**		**0.00**	**1.01**	**1.01**
**State-level variable**									
direct access specialist	**0.930**	**0.00**	**0.886**	**0.974**	**0.91**		**0.00**	**0.88**	**0.94**
**Variance components**									
Level 1 (person) *	3.2899					3.2899			
Level 2 (county)	0.01032					0.01068			
Level 3 (state)	0.00375					0.00327			

* For logistic multilevel models, the variance for level one (person level) is assumed to be π^2^/3. Shaded cells represent rows with no predictor included in the model specification. This person-centered isolation estimate (Model 2) is likely dominated by segregated Whites due to the fact that the vast majority (77%) of the study sample is White, and the White population is highly segregated (Figure 2). These estimates are national average effects and are cross-sectional, thus causality cannot be determined. Bold numbers indicate statistical significance.

## Abbreviations

BC	breast cancer
ACS	American Cancer Society
CDC	Centers for Disease Control and Prevention
GLLAMM	generalized linear latent and mixed models
NCSL	National Conference of State Legislators
NIH	National Institutes of Health
RDC	research data center
USCS	U.S. Cancer Statistics
